# Diffusion and functional MRI reveal microstructural and network connectivity impairment in adult-onset neuronal intranuclear inclusion disease

**DOI:** 10.3389/fnagi.2024.1478065

**Published:** 2024-10-11

**Authors:** Rui Zhu, Junyu Qu, Yongsheng Wu, Guihua Xu, Dawei Wang

**Affiliations:** ^1^Department of Radiology, Qilu Hospital of Shandong University, Qilu Medical Imaging Institute of Shandong University, Jinan, China; ^2^Research Institute of Shandong University, Magnetic Field-free Medicine and Functional Imaging, Jinan, China; ^3^Shandong Key Laboratory, Magnetic Field-free Medicine and Functional Imaging (MF), Jinan, China

**Keywords:** neuronal intranuclear inclusion disease, diffusion kurtosis imaging, functional connectivity, cognitive decline, neuroimaging biomarkers

## Abstract

**Objectives:**

Neuronal intranuclear inclusion disease (NIID) is a rare neurodegenerative disorder lacking reliable neuroimaging biomarkers. This study aimed to evaluate microstructural and functional connectivity alterations using diffusion kurtosis imaging (DKI) and resting-state fMRI (rs-fMRI), and to investigate their diagnostic potential as biomarkers.

**Methods:**

Twenty-three patients with NIID and 40 matched healthy controls (HCs) were recruited. Firstly, gray matter (GM) and white matter (WM) changes were assessed by voxel-based analysis (VBA) and tract-based spatial statistics (TBSS). Then we explored modifications in brain functional networks connectivity by independent component analysis. And the relationship between the altered DKI parameters and neuropsychological evaluation was analyzed. Finally, a receiver operating characteristic (ROC) curve was used to evaluate the diagnostic performance of different gray matter and white matter parameters.

**Results:**

Compared with the HCs, NIID patients showed reduced mean kurtosis (MK), radial kurtosis (RK), axial kurtosis (AK), and kurtosis fractional anisotropy (KFA) values in deep gray matter regions. Significantly decreased MK, RK, AK, KFA and fractional anisotropy (FA), and increased mean diffusivity (MD) values were observed in extensive white matter fiber tracts. Notable alterations in functional connectivity were also detected. Among all DKI parameters, the diagnostic efficiency of AK in GM and FA in WM regions was the highest.

**Conclusion:**

Adult-onset NIID patients exhibited altered microstructure and functional network connectivity. Our findings suggest that DKI parameters may serve as potential imaging biomarkers for diagnosing adult-onset NIID.

## Introduction

Neuronal intranuclear inclusion disease (NIID) is a rare neurodegenerative disease, primarily reported in East Asia but increasingly recognized in Western countries (Liu et al., 2022). It is characterized by the presence of eosinophilic intranuclear inclusions within cells of the central, peripheral, and autonomic nervous systems, as well as in various visceral organs (Sone et al., 2016). NIID is classified into infantile, adolescent, and adult forms. The adult form of NIID is further subdivided into familial and disseminated types according to genetic characteristics (Sone et al., 2016). The clinical manifestations of adult-onset NIID are significantly heterogeneous, encompassing cognitive impairment, paroxysmal symptoms, parkinsonism, encephalitic episodes, dysuria symptoms, and cerebellar ataxia (Sone et al., 2016; Tian et al., 2022; Bao et al., 2023). Of these, cognitive impairment represents the most prevalent symptom, with a reported occurrence rate of 94.7% among patients with disseminated adult-onset NIID (Sone et al., 2016).

In recent years, the number of reported NIID cases has significantly increased due to the application of skin biopsies and the discovery of the *NOTCH2NLC* gene (Sone et al., 2011; Sone et al., 2016; Liu et al., 2022; Tian et al., 2022). A characteristic MRI feature is the high signal intensity at the corticomedullary junction on diffusion-weighted imaging (DWI), which provides a strong indication for a further skin biopsy (Sone et al., 2016) and genetic examination (Tian et al., 2019) to confirm the diagnosis in suspected patients. Some patients also exhibit extensive white matter hyperintensity (WMH) on T2-weighted/fluid-attenuated inversion recovery (FLAIR) images (Liu et al., 2019; Bao et al., 2023), and leukoencephalopathy is correlated to the cognitive impairment in adult-onset NIID(Wang et al., 2021). Neuroimaging serves as a noninvasive and the most accessible screening and diagnostic tool. However, some NIID patients do not exhibit the typical features on DWI (Okamura et al., 2020; Wang et al., 2022). Therefore, there is a pressing need for more precise imaging biomarkers to facilitate diagnosis of NIID and to better understand its underlying mechanisms.

Diffusion kurtosis imaging (DKI) is an advanced diffusion MRI technique that captures both diffusion tensor imaging (DTI) parameters, such as fractional anisotropy (FA) and mean diffusivity (MD) and DKI-specific parameters including mean kurtosis (MK), radial kurtosis (RK), axial kurtosis (AK), and kurtosis fractional anisotropy (KFA) (Jensen and Helpern, 2010; Wu and Cheung, 2010). This method significantly enhances the detection of subtle changes in the brain’s microstructure and establishes correlations with disease severity (Steven et al., 2014; Arab et al., 2018; Falk Delgado et al., 2018; Shi et al., 2019; Zheng et al., 2020). Additionally, resting-state functional magnetic resonance imaging (rs-fMRI) assesses the temporal correlations between cortical oxygenation and metabolic activities, examines the consistency and coordination among different brain regions, and evaluates changes in brain networks from a functional standpoint (Raimondo et al., 2021). The impact of damage to gray matter (GM) and white matter (WM) fibers, or abnormalities in functional connectivity, has been shown to be profoundly associated with cognitive impairments in various neurodegenerative diseases, such as Alzheimer’s disease (AD) and Parkinson’s disease (PD) (Raj et al., 2022; Bergamino et al., 2024; Huang et al., 2024).

Currently, comprehensive research on NIID is still insufficient. Since the condition was first identified in 1968 (Lindenberg et al., 1968), fewer than 700 cases have been documented in the literature, with fewer than 100 of these cases presenting comprehensive imaging characteristics (Zhu et al., 2024). Previous MRI studies have primarily been case reports with the primary emphasis on conventional imaging characteristics, and have not conducted an exhaustive and systematic analysis of the complex microstructural characteristics of NIID. What’s more, the specific ways in which the brain changes in adult-onset NIID and how these changes affect cognitive performance are yet to be fully explored.

In our study, we aimed to: (1) assess alterations in the global GM using voxel-based analysis (VBA) and evaluate changes in the WM microstructure through tract-based spatial statistics (TBSS) analysis of NIID patients, (2) explore modifications in brain functional networks connectivity by means of independent component analysis (ICA), and (3) analyze the potential relationship between specific microstructural changes and neuropsychological assessments, as well as explore the diagnostic performances of DKI parameters for patients with NIID. Through this study, we hope to promote a deeper understanding of the pathological mechanisms of NIID, and facilitate development of potential imaging biomarkers for the clinical diagnosis of adult-onset NIID.

## Materials and methods

### General information

A total of 23 adult-onset NIID patients were retrospectively enrolled from the Qilu Hospital of Shandong University between January 2019 and October 2023. The inclusion criteria for adult-onset NIID patients were as follows: (1) age of 18 years or older; (2) a confirmed diagnosis based on positive results from a skin pathology and/or analysis of the *NOTCH2NLC* gene; (3) stable vital signs, with the willingness and ability to undergo MRI examinations (Sone et al., 2016). The following criteria were used to exclude subjects from the study: (1) evidence of cerebral infarction, lacunar infarcts, or intracerebral hemorrhage on MRI, or a history of neurological or psychiatric disorders; (2) presence of leukoencephalopathies attributed to demyelinating diseases, toxin exposure, infection, or neoplasms; (3) contraindications for MRI scans. Forty healthy controls (HCs) matched for age, gender, and education level were also included using the same exclusion criteria as the patients during the same period. All of the 23 patients underwent Mini-Mental State Examination (MMSE) and the Montreal Cognitive Assessment (MoCA) tests by at least two experienced neurologists. Prior to participation, all subjects or their legal guardians provided written informed consent, and the study was approved by the Medical Ethics Committee of Qilu Hospital of Shandong University.

### MRI protocols

MRI was conducted on a 3.0-Tesla magnetic field scanner (Siemens Erlangen, Germany) utilizing a standard 20-channel head receiver coil. Participants were informed that they should keep their eyes closed and remain still throughout the scanning process. T2WI was performed on all subjects to exclude intracranial lesions, followed by 3D-T1WI sagittal high-resolution, DTI sequence, DKI sequence, and resting-state functional MRI scans. T1-weighted anatomical reference images were acquired using an MPRAGE sequence with the following parameters: repetition time /echo time = 2000/2.3 ms, resolution = 1 × 1 × 1 mm^3^, matrix size = 192 × 256 × 256, inversion time (TI) = 900 ms, iPAT = 2, and bandwidth = 190 Hz. DTI images were acquired in the axial plane using the single shot-echo planar imaging technique with the following parameters: b values = 0, 1,000 s/mm^2^; diffusion direction = 64, TR/TE = 6400/98 ms, field of view (FOV) = 256 × 256 mm^2^, resolution = 2 × 2 mm^2^, slices = 45, slice thickness = 3 mm, and bandwidth = 1,502 Hz. DKI images were obtained with three b-values (b = 0, 1,000, and 2000s/mm^2^) along 20 gradient directions for each non-zero b value using echo planar imaging (EPI) sequence: TR/TE = 9600/96 ms, FOV = 256 × 256 mm^2^, voxel size = 2.0 × 2.0 × 3.0 mm^3^, slices = 45, slice thickness = 3 mm. The resting-state functional MRI scan sequence used a gradient EPI sequence: TR/TE = 2000/30 ms, flip angle = 90°, FOV = 256 × 256 mm^2^, slice thickness = 3 mm, and 180 time points were collected with a voxel size of 3.4 × 3.4 × 3.0 mm^3^.

### Diffusion data processing

The image processing primarily involved initial preprocessing and the computation of diffusion metrics. The process began with the conversion of all diffusion MRI data from DICOM to NIFTI format using the dcm2niigui tool. The FSL software^1^ was utilized to correct for motion and eddy currents and to remove the scalp and skull. A rigorous quality control assessment ensured the exclusion of data compromised by significant deformations, artifacts, or excessive noise. DTI metrics, specifically FA and MD, were calculated using the dtifit command of FSL. Additionally, DKI parameters including MK, RK, AK, KFA, FA, and MD were derived via the Diffusion Kurtosis Estimator^2^ software.

We analyzed DKI parameters such as MK, RK, AK, and KFA in the gray matter at the voxel level. Using SPM12,^3^ structural images were segmented into gray matter, white matter, and cerebrospinal fluid, and subsequently aligned to the Montreal Neurological Institute (MNI) standard space. This alignment integrated the parameters into DKI maps, which then underwent spatial smoothing with a full width at half maximum (FWHM) of 6 mm. The REST software^4^ computed average gray matter images for the healthy controls group, while mricron was employed to create gray matter masks, facilitating voxel-wise evaluations within these regions. Group differences in DKI maps were statistically analyzed via a two-sample t-test using SPM12, adjusting for family-wise error (FWE) with age and gender as covariates. Significance was defined as a voxel-level *p* value <0.05 with a minimum cluster size of 100.

For TBSS analysis of white matter, non-linear co-registration using FSL was employed to align DTI-FA or DKI-FA maps to the FMRIB58_FA_1mm standard (Jenkinson et al., 2012). This process involved creating an average FA skeleton, applying a threshold of 0.2 (Taylor et al., 2016), and projecting the FA maps onto the mean skeleton. The derived registration and projection data from the FA analysis were subsequently applied to other DTI and DKI metrics, respectively. A general linear model with age and gender as covariates was used to compare data between NIID patients and HCs. White matter fiber skeletons from TBSS served as the mask for voxel-wise statistical analyses, where significance was determined using a *p* value <0.05 (two-tailed), corrected for FWE through the threshold-free cluster enhancement (TFCE) method in FSL. Visualization enhancements were achieved using the tbss_fill script. Using the DPABI software package in Matlab 2016b, parameter maps standardized to MNI space were analyzed to extract relevant parameter values from brain regions showing differences in fiber bundle parameters between the two groups, utilizing the JHU ICBM-DTI-81 White-Matter Labels as a template. Based on the parameter maps standardized to MNI space, the DPABI software package in Matlab 2016b was utilized to extract the relevant parameter values of the brain regions where there were differences in fiber bundle parameters between the two groups using JHU ICBM-DTI-81 White-Matter Labels as a template. To quantitatively assess the sensitivity of parameters derived from DKI and DTI in detecting impairments in NIID patients’ brain tissue integrity, we calculated the percentage of abnormal voxels. This was done relative to the total number of voxels in the entire skeleton for each parameter.

### Independent component analysis

The image preprocessing was conducted using the Resting-State fMRI Data Analysis Toolkit.^5^ After completing the preprocessing of fMRI images, the Group ICA of fMRI Toolbox^6^ was used for ICA. This toolbox decomposes the data into statistically independent time components with non-Gaussian distribution and their corresponding spatial components in a linear combination. The number of components was estimated using the minimum description length (MDL) algorithm, and the dimensionality of the fMRI images was reduced using two rounds of principal component analysis (PCA). Blind source separation was then performed on the reduced fMRI data at the group level using the default network templates provided by GIFT. The default network components and corresponding time series were identified at the group level using multiple regression. Finally, the individual-level default network components were obtained through back-reconstruction and converted into Z-scores for further analysis. The informax algorithm was used to perform 100 iterations of ICA, decomposing each subject’s fMRI images into 20 spatially independent components. Ten meaningful brain networks, identified as classic resting-state networks (RSNs) in terms of anatomy and function, were extracted through visual inspection. Statistical analysis was then performed on the meaningful spatially independent components of all subjects. A two-sample t-test was used to examine changes within and between functional connectivity networks between the NIID and HCs groups. A *p* value <0.05 was considered statistically significant.

### Statistical analysis

Data analysis was performed using SPSS 26.0 statistical software. The normality of all quantitative data was evaluated using the Shapiro–Wilk test. Data with a normal distribution were described as mean ± standard deviation (x ± s) and analyzed using t-tests. Qualitative data were presented as frequencies and analyzed using the chi-square test to identify any significant differences.

Statistically significant DKI parameter values in gray matter brain regions and fiber tracts were correlated with the MMSE and MoCA scores. The significance level was set at *p* value <0.05, and results were corrected for multiple comparisons using the Bonferroni method. Pearson’s correlation analysis was employed for data that conformed to a normal distribution, while Spearman’s correlation analysis was used for non-normally distributed data to obtain the correlation coefficient (*r*).

Receiver operating characteristic (ROC) curves were used to analyze mean DKI values in GM and WM regions that showed statistically significant difference in patients with NIID, respectively. Diagnostic accuracy was indicated by the area under the ROC curve (AUC). Furthermore, a binary logistic regression model was constructed to explore the overall diagnostic efficacy of combining MK, RK, AK, and KFA metrics in both GM and WM regions.

## Results

### Demographic and clinical characteristics of the subjects

This study had 63 participants in total, including 23 patients with NIID (61.57 ± 6.04 years, 6 males) and 40 HCs (64.03 ± 4.20 years, 12 males). No statistically significant differences in age, gender, or education level between the NIID and HCs groups (*p* = 0.093, *p* = 0.741, *p* = 0.096, respectively). The MMSE and MoCA scores of NIID patients were lower than those of HCs group (*p* < 0.001). Among the 23 adult-onset NIID patients, 16 subjects underwent *NOTCH2NLC* GGC gene testing. The median size of the expanded *NOTCH2NLC* GGC repeats in the 16 NIID patients was 113.5 (range 91–191). The detailed demographics and clinical data are displayed in Table 1.

**Table 1:** Demographic and clinical characteristics of patients with NIID and HCs.

Variable	NIID (*n* = 23)	HCs (*n* = 40)	*t/χ*^2^ value	*p* value
Age(years)	61.57 ± 6.04	64.03 ± 4.20	−1.726	0.093*
Gender(n)			0.110	0.741^†^
Male	6(26.1%)	12(30.0%)		
Female	17(73.9%)	28(70.0%)		
Education level(n)			4.689	0.096^†^
Primary and below	9(39.1%)	6(15.0%)		
Middle school	12(52.2%)	29(72.5%)		
University and above	2(8.7%)	5(12.5%)		
MMSE(scores)	22.87 ± 4.67	28.98 ± 1.23	−6.144	<0.001*
MoCA(scores)	17.09 ± 5.60	27.35 ± 1.19	−8.678	<0.001*
*NOTCH2NLC* GGC repeat size^#^	113.5 (91–191)			
Disease duration (years)	2.5 ± 0.5			

Data are presented as mean ± standard or number of participants (percentage) deviation or medians (ranges). *Two-sample *t* test, ^†^Chi-squared test, ^#^7 patients had no data on NOTCH2NLC GGC repeat size. NIID, Neuronal Intranuclear Inclusion Disease; HCs, Healthy Controls; MMSE, Mini-Mental State Examination; MoCA, Montreal Cognitive Assessment.

### Gray matter alterations of DKI parameters in NIID patients

**Figure 1:**
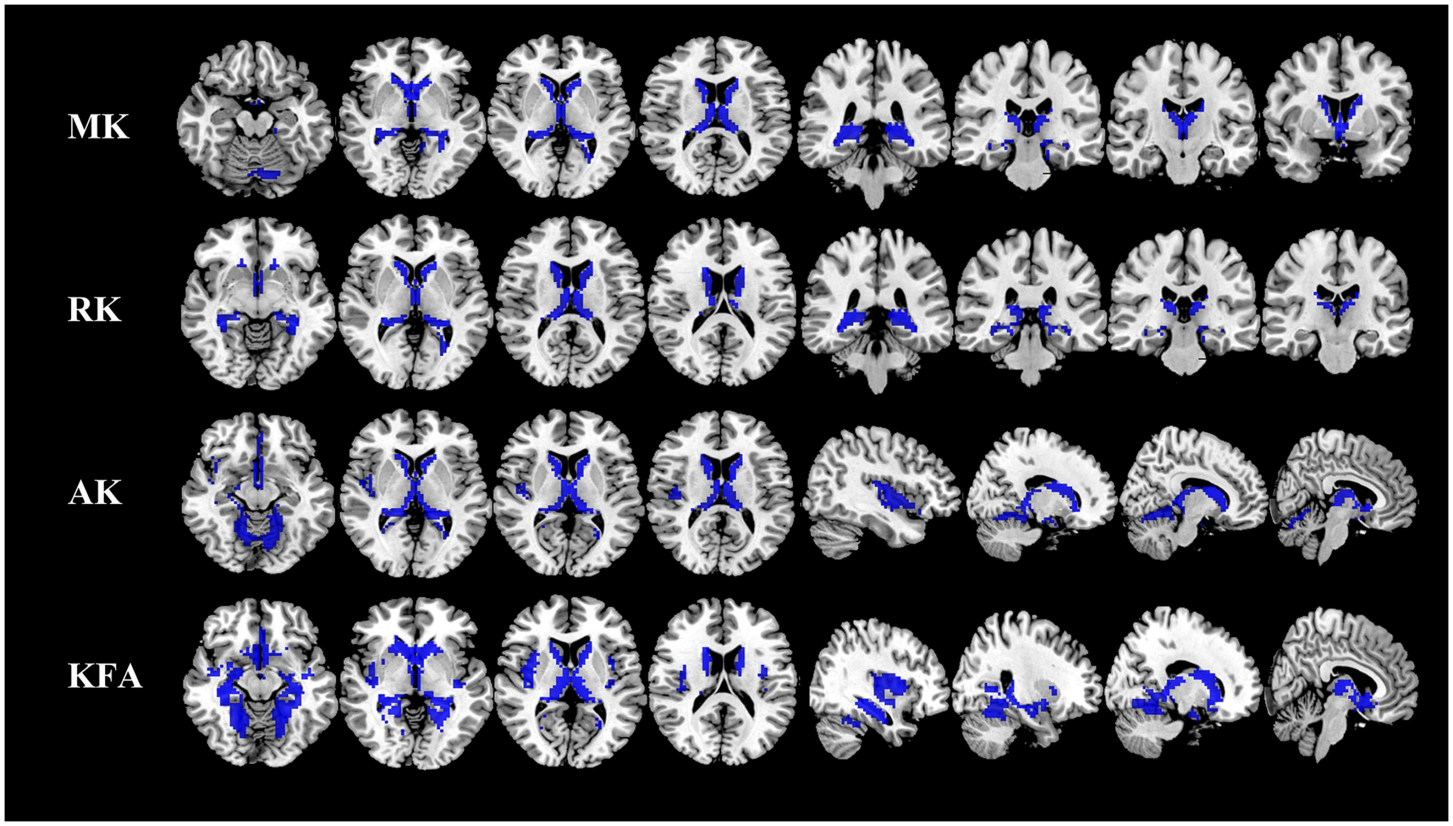
Comparison of DKI metrics in gray matter between NIID patients and HCs. Voxel-based analysis shows gray matter regions with significant (*p* < 0.05 and cluster size ≥100, family wise error corrected) differences in the MK, RK, AK, and KFA between NIID patients and HCs. Blue represents decreased regions in NIID patients. DKI, Diffusion Kurtosis Imaging; NIID, Neuronal Intranuclear Inclusion Disease; HCs, Healthy Controls; MK, Mean Kurtosis; RK, Radial Kurtosis; AK, Axial Kurtosis; KFA, Kurtosis Fractional Anisotropy.

Compared to the HCs, the NIID patients demonstrated significant decreases in DKI parameters across various gray matter regions using voxel-based analysis (Figure 1; Table 2). For MK, reductions were found in bilateral regions including the caudate, thalamus, and hippocampus. RK also showed decreases in these same areas. AK measurements indicated similar decreases but included additional reductions in the cerebellum (areas 6 and 4_5) and the lingual gyrus. Notably, the significant KFA decrease were investigated mostly in the caudate, hippocampus, insula, thalamus, fusiform gyrus, cerebellum, and para-hippocampal regions.

**Table 2:** Gray matter regions with significantly decreased DKI parameters in the NIID patients.

Indexes	Cluster size	*t* value	Peak MNI coordinate			Major brain regions
			X	Y	Z	(AAL)
MK						
01	1,038	−10.2018	0	9	−3	Caudate _L, Caudate _R
						Thalamus _L, Thalamus _R
						Hippocampus _L, Hippocampus _R
02	149	−6.8428	−9	−75	−15	Cerebelum_6_L
RK						
01	976	−10.7450	0	9	−3	Caudate _L, Caudate _R
						Thalamus _L, Thalamus _R
						Hippocampus _L, Hippocampus _R
AK						
01	1,709	−10.8672	0	15	−3	Caudate _L, Caudate _R
						Thalamus _L, Thalamus _R
						Hippocampus _L, Hippocampus _R
						Cerebelum_6_L, Cerebelum_4_5_R
						Lingual _L, Lingual _R
02	136	−7.2276	42	−15	−3	Insula _R
KFA						
01	2,949	−13.1636	12	18	−6	Caudate _L, Caudate _R
						Hippocampus _L, Hippocampus _R
						Lingual _L, Lingual _R
						Insula _R, Insula _L
						Thalamus _L, Thalamus _R
						Fusiform _L, Fusiform _R
						Cerebelum_6_R, Cerebelum_4_5_R
						Para-Hippocampal _R, Para-Hippocampal _L

Corrected by family wise error (FWE) criterion and set at *p* < 0.05, cluster size ≥ 100. DKI, Diffusion Kurtosis Imaging; MNI, Montreal Neurological Institute; AAL, Anatomical Automatic Labeling; MK, Mean Kurtosis; RK, Radial Kurtosis; AK, Axial Kurtosis; KFA, Kurtosis Fractional Anisotropy.

### White matter changes of DTI and DKI parameters in NIID patients

**Figure 2:**
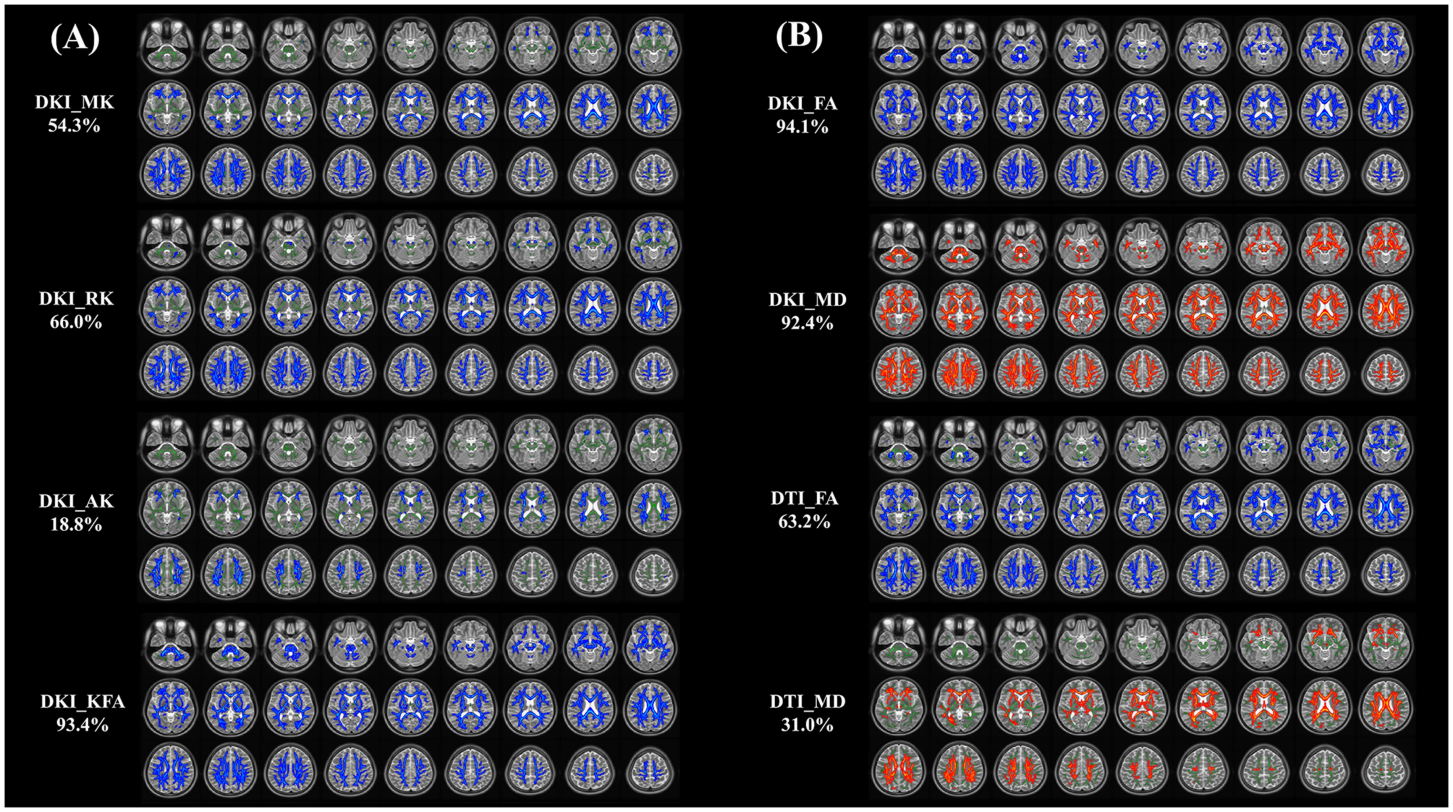
**(A, B)** Tract-based spatial statistics (TBSS) results of DKI and DTI metrics between NIID and HCs groups. This TBSS analysis highlights significant differences (*p* < 0.05, threshold-free cluster enhancement corrected) in both DKI metrics (MK, RK, AK, KFA, FA, and MD) and DTI metrics (FA, MD) across white matter regions between NIID patients and HCs. The background of results is the mean FA skeleton (green) of all participants. Blue denotes reduction and red represents increase in NIID patients. The percentage in the left column represents the percentage of the abnormal voxels relative to the whole skeleton voxels for each parameter. DKI, Diffusion Kurtosis Imaging; DTI, Diffusion Tensor Imaging; NIID, Neuronal Intranuclear Inclusion Disease; HCs, Healthy Controls; MK, Mean Kurtosis; RK, Radial Kurtosis; AK, Axial Kurtosis; KFA, Kurtosis Fractional Anisotropy; FA, Fractional Anisotropy; MD, Mean Diffusivity.

The whole-brain TBSS analysis of DKI and DTI parameters revealed significantly lower MK, RK, AK, KFA and FA, and higher MD values in patients with NIID than the HCs (*p* < 0.05, two-tailed, TFCE corrected, Supplementary Table S1). The decreased DKI parameters of NIID patients were shown in extensive WM regions, mainly including the association fibers (such as superior longitudinal fasciculus, uncinate fasciculus, and superior fronto-occipital fasciculus), the commissural fibers (such as corpus callosum), and the projection fibers (such as corona radiata, internal capsule, posterior thalamic radiation, and cerebral peduncle) (Table 3). DKI-MK, DKI-RK, DKI-AK, and DKI-KFA could detect abnormal diffusion in 54.3, 66.0, 18.8, and 93.4% of voxels of the whole WM skeleton, respectively (Figure 2A). Interestingly, DKI-derived map showed more brain areas in the white matter with decreased FA and increased MD compared to DTI map. DKI-FA and DKI-MD could detect abnormal diffusion in 94.1 and 92.4%, respectively, of voxels in the whole WM skeleton, while DTI-FA and DTI-MD were 63.2 and 31.0% (Figure 2B).

**Table 3:** White matter tracts with significantly different DKI parameters between the two groups in the JHU atlas.

Fiber Tracts	MK	RK	AK	KFA	FA	MD
Middle cerebellar peduncle	–	Y	–	Y	Y	Y
Pontine crossing tract	–	–	–	Y	Y	Y
Corpus callosum	Y	Y	Y	Y	Y	Y
Fornix	Y	Y	Y	-	-	Y
Corticospinal tract	-	B	–	B	B	B
Medial lemniscus	–	–	–	B	B	B
Inferior cerebellar peduncle	–	–	–	B	B	B
Superior cerebellar peduncle	–	–	–	B	B	B
Cerebral peduncle	–	B	–	B	B	B
Anterior limb of internal capsule	B	B	B	B	B	B
Posterior limb of internal capsule	B	B	B	B	B	B
Retrolenticular part of internal capsule	B	B	B	B	B	B
Anterior corona radiata	B	B	B	B	B	B
Superior corona radiata	B	B	B	B	B	B
Posterior corona radiata	B	B	B	B	B	B
Posterior thalamic radiation	B	B	B	B	B	B
Sagittal stratum	L	B	R	B	B	B
External capsule	B	B	B	B	B	B
Cingulum (cingulate gyrus)	B	B	–	B	B	B
Cingulum (hippocampus)	–	R	–	B	B	B
Fornix-Stria terminalis	R	B	L	B	B	B
Superior longitudinal fasciculus	B	B	B	B	B	B
Superior fronto-occipital-fasciculus	B	B	B	B	B	B
Uncinate fasciculus	–	–	–	B	B	B
Tapetum	B	B	B	B	B	B

JHU ICBM-DTI-81 White-Matter atlas consists 48 white matter regions (left and right). The R, L and B represent fiber tract microstructural changes in the right, left and bilateral side. Y means damage in the fiber tracts. Corrected by family wise error (FWE) using the threshold-free cluster enhancement (TFCE) and set at *p* < 0.05 (two-tailed). DKI, Diffusion Kurtosis Imaging; MK, Mean Kurtosis; RK, Radial Kurtosis; AK, Axial Kurtosis; KFA, Kurtosis Fractional Anisotropy; FA, Fractional Anisotropy; MD, Mean Diffusivity; R, Right; L, Left; B, Bilateral; Y, Yes.

### Changes in functional connectivity of brain networks between the two groups

**Figure 3:**
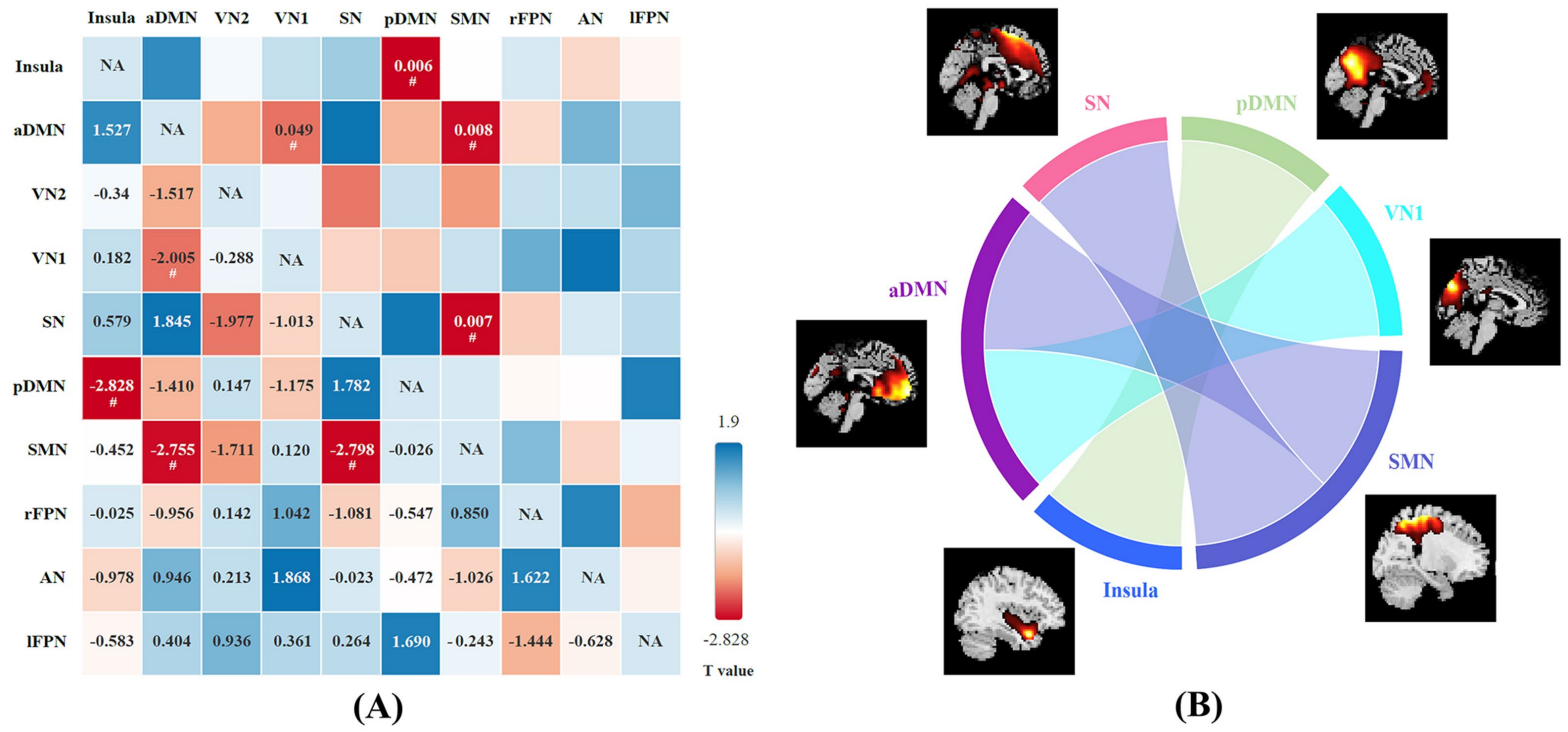
(A) The difference in functional network connectivity (FNC) between NIID patients and healthy controls is statistically significant (two-sample t-test, ^#^*p* < 0.05). The *t*-values are located in the lower left region, while the significant *p*-values are situated in the upper right region. Blue checks indicate positive correlations, while red checks indicate negative correlations. The intensity of the color (lighter or darker shades) reflects the absolute value of the correlation coefficient, with more intense colors indicating stronger correlations. (B) Group differences of FNC in connectogram (two-sample t-test, *p* < 0.05).

A total of 20 independent components were discovered through ICA. Ten meaningful RSNs were extracted from all the patients, including the insula, anterior default mode network (aDMN), visual network (VN1 and VN2), salience network (SN), posterior default mode network (pDMN), sensorimotor network (SMN), right frontoparietal network (rFPN), left frontoparietal network (lFPN), and auditory network (AN). Compared with the HCs, NIID patients show significantly decreased connections between the aDMN and VN1, the pDMN and Insula, the aDMN and SMN, the SMN and SN (*p* < 0.05; Figure 3). No significant differences were found in functional connectivity within the networks of the two groups.

### Correlation between DKI metrics and neuropsychological assessments

**Figure 4:**
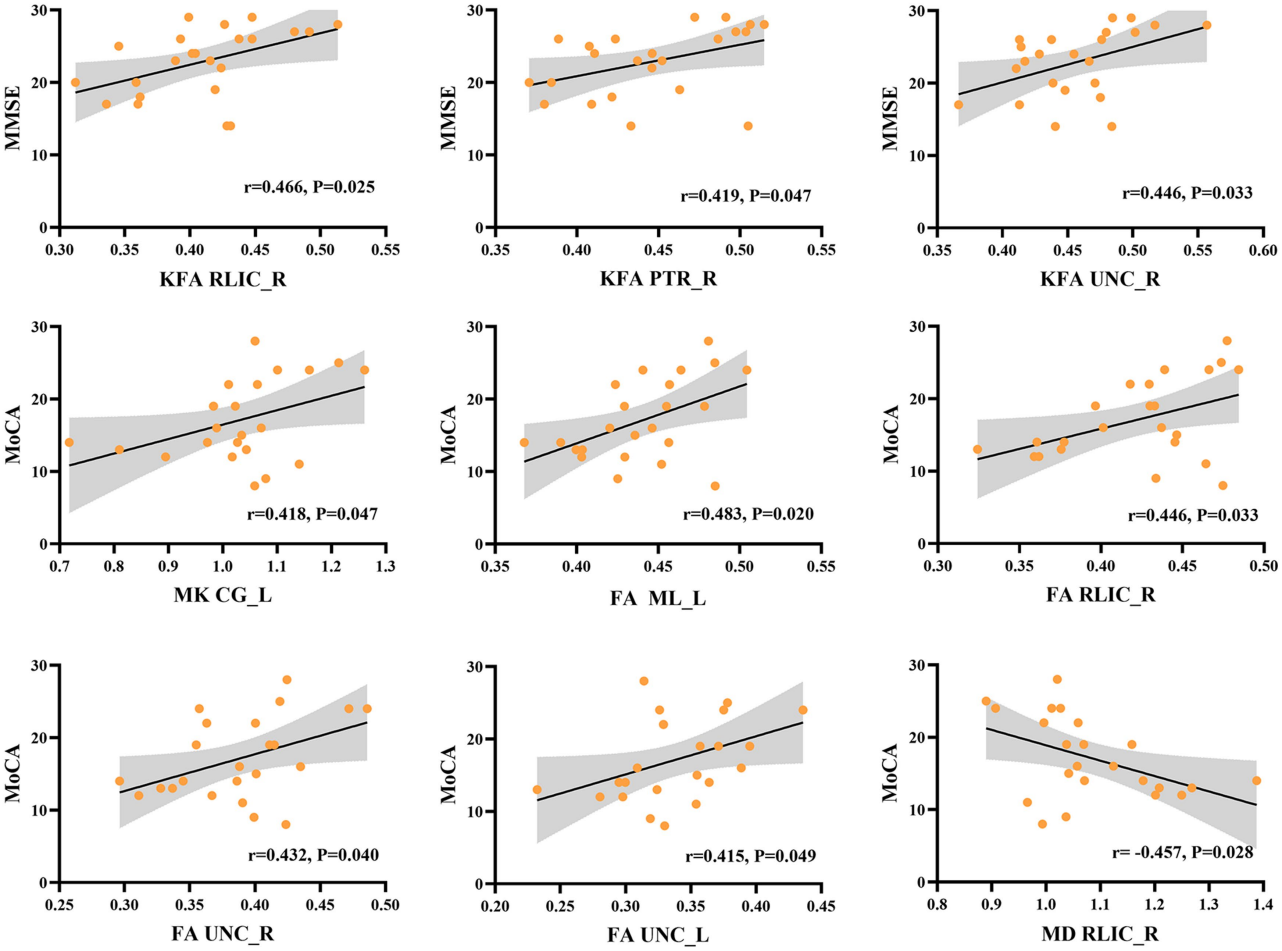
Correlation between DKI parameters in specific white matter tracts and the neuropsychological assessments in NIID group. KFA in right retrolenticular part of internal capsule (RLIC), right posterior thalamic radiation (PTR), and right uncinate fasciculus (UNC) correlated positively with the Mini-Mental State Examination (MMSE) scores. MK in light cingulate gyrus (CG), FA in left medial lemniscus (ML), right RLIC, right and left UNC correlated positively with Montreal Cognitive Assessment (MoCA) scores. Besides, MD in right RLIC showed negative correlations with MoCA scores.

Our correlational analysis revealed that neuropsychological assessments (as measured by MMSE and MoCA scores) were associated with DKI parameters in certain WM regions, while no significant correlations were found with DKI parameters in GM regions (*p* > 0.05). Regarding DKI-derived kurtosis parameters in WM regions, the mean KFA values in the right retrolenticular part of internal capsule (RLIC) (*r* = 0.466, *p* = 0.025), posterior thalamic radiation (PTR) (*r* = 0.419, *p* = 0.047), and uncinate fasciculus (UNC) (*r* = 0.446, *p* = 0.033) all showed significantly positive correlations with MMSE scores. The mean MK values in the light cingulate gyrus (CG) (*r* = 0.418, *p* = 0.047) showed significantly positive correlations with MoCA scores. Regarding DKI-derived diffusion parameters in WM regions, the mean FA values in the left medial lemniscus (ML) (*r* = 0.483, *p* = 0.020), right RLIC (*r* = 0.446, *p* = 0.033), right UNC (*r* = 0.432, *p* = 0.040), and left UNC (*r* = 0.415, *p* = 0.049) showed significantly positive correlations with MoCA, while the mean MD values in right RLIC (*r* = − 0.457, *p* = 0.028) showed negative correlations with MoCA scores (Figure 4).

### Relationship between the copy numbers of NOTCH2NLC GGC and DKI parameters

We further explored the relationship between the copy numbers of *NOTCH2NLC* GGC and DKI parameters in abnormal brain regions of gray matter and white matter. However, no significant correlation was identified between GM_MK, GM_RK, GM_AK, GM_KFA, WM_MK, WM_RK, WM_AK, WM_KFA, WM_FA, WM_MD values and the number of GGC amplifications of the *NOTCH2NLC* gene (Supplementary Table S2).

### The ROC analyses for diagnostic performances

**Figure 5:**
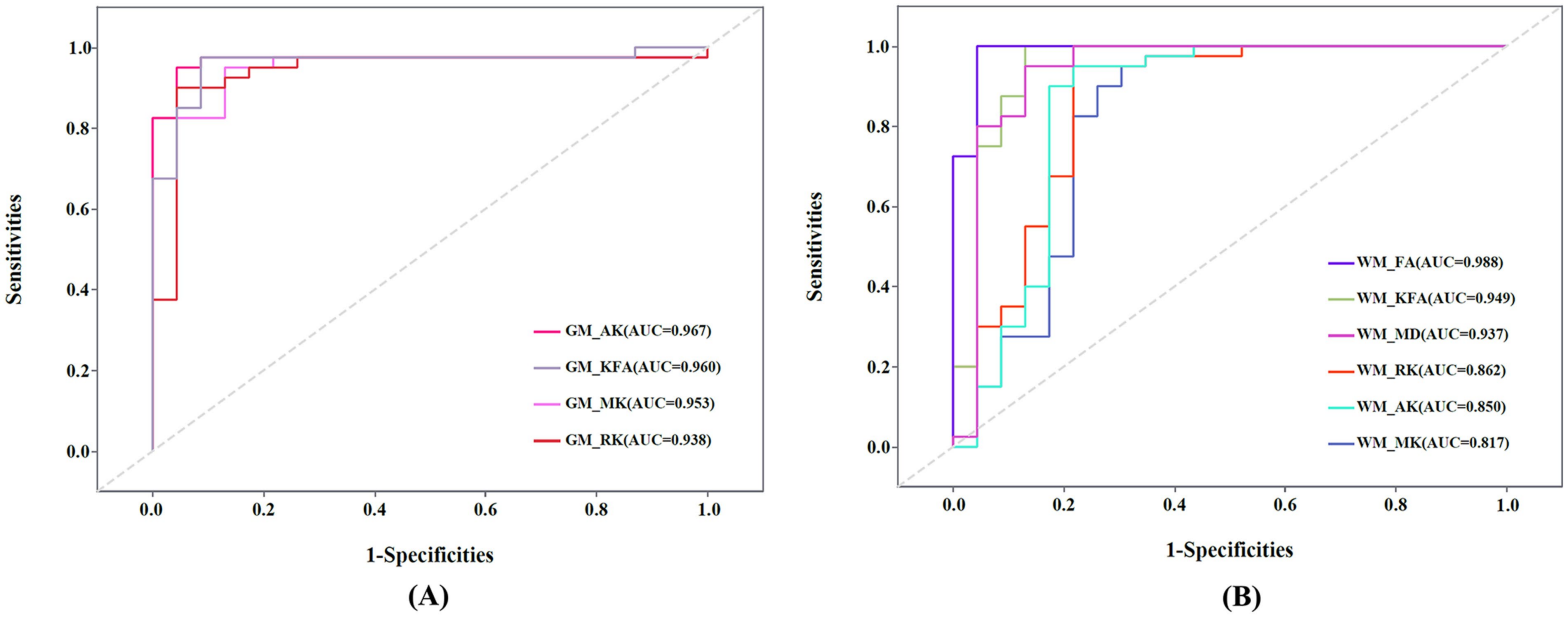
(A) Receiver operating characteristic (ROC) curve of mean kurtosis (MK), radial kurtosis (RK), axial kurtosis (AK), and kurtosis fractional anisotropy (KFA) in gray matter (GM) regions. (B) ROC curve of MK, RK, AK, KFA, fractional anisotropy (FA), and mean diffusivity (MD) in white matter (WM) regions. AUC: Areas under the ROC curve.

The ROC curve of the DKI parameters in gray matter and white matter regions is shown in Figure 5. In GM regions, the diagnostic efficiency of AK (AUC = 0.967) was the most promising, followed by KFA (AUC = 0.960). In WM regions, the diagnostic efficiency of FA (AUC = 0.988) was the most promising, followed by KFA (AUC = 0.949). The AUCs of the MK, RK, and AK parameters in the combined GM and WM regions were higher than their AUCs in the WM region (AUC_combined_MK_ vs. AUC_WM_MK_ = 0.952 vs. 0.817, AUC_combined_RK_ vs. AUC_WM_RK_ = 0.935 vs. 0.862, AUC_combined_AK_ vs. AUC_WM_AK_ = 0.964 vs. 0.850), and lower than their AUCs in the GM region (AUC_combined_MK_ vs. AUC_GM_MK_ = 0.952 vs. 0.953, AUC_combined_RK_ vs. AUC_GM_RK_ = 0.935 vs. 0.938, AUC_combined_AK_ vs. AUC_GM_AK_ = 0.964 vs. 0.967) (Supplementary Figures 1A,C,D). The AUCs of the KFA parameter in the combined regions were higher than its AUCs in both the WM and GM regions (AUC_combined_KFA_ vs. AUC_GM_KFA_ vs. AUC_WM_KFA_ = 0.962 vs. 0.960 vs. 0.949) (Supplementary Figure 1B). However, this differences in diagnostic efficacy between combined and separate regions were not statistically significant (all *p* > 0.05).

## Discussion

To the best of our knowledge, this study represents the pioneering effort to investigate alterations in microstructure and functional connectivity in adult-onset NIID patients by employing DKI and rs-fMRI. Our research produced five major findings and implications: (1) NIID patients had reduced MK, RK, AK and KFA values in several important deep gray matter regions, suggesting the presence of microstructural impairment. (2) NIID patients demonstrated extensive damage in the WM fiber tracts, and DKI map manifested more damaged WM areas than DTI map. (3) The KFA, MK, FA, and MD values in several key cognitive regions of white matter were significantly correlated with the MMSE and MoCA scores, demonstrating that microstructural abnormalities of specific WM fiber tracts are associated with cognitive decline. (4) We identified reduced functional connectivity in several brain networks in NIID patients, which helps to better explain their clinical manifestations and pathological mechanisms. (5) DKI parameters has high diagnostic efficacy and can be used as neuroimaging biomarkers for the accurate diagnosis of NIID.

DKI has been widely used to assess the microstructural changes of white matter and gray matter (Xiong et al., 2019; Cheung et al., 2009). DKI indices are instrumental in delineating structural complexities, influenced by factors such as glial activity and the integrity of synapses, neurites, and myelinated axons (Xiong et al., 2019; Steven et al., 2014). In our study, we observed reduced values of MK, RK, AK and KFA primarily in the caudate, thalamus, and hippocampus, which may reflect neurodegenerative alterations or the loss of neurons (Louis, 2016). Previous studies have demonstrated that AD patients show decreased DKI parameters in the deep gray matter of the brain (Raj et al., 2022; Niu et al., 2023). Consequently, such microstructural alterations in gray matter may also be a potential mechanism for cognitive deficits in patients with NIID. Notably, we found AK was the most sensitive metric for capturing the loss of gray matter microstructural integrity, distinguishing NIID patients from HCs among the four DKI metrics.

Liu et al. demonstrated severe cerebral white matter degeneration, demyelination of white matter fibers, and axonal damage in adult patients with NIID by autopsy (Liu et al., 2008). A previous case report documented the occurrence of DTI in patients with NIID, presenting with white matter bundle lesions (Liu et al., 2019). In our study, we found extensive microstructural destruction of white matter fiber tracts in a cohort of patients with NIID. KFA, a sensitive parameter indicating anisotropy, measures the compactness and regularity of structured organization (Gupta et al., 2022). It has been reported that the decreased anisotropy values are associated with the lysis of cells, disruption of fibrous, and the loss of tissue (Wu et al., 2020). Consequently, we surmised that the diminished KFA values of extensive white matter fiber bundles observed in NIID patients may also be associated with their microstructural disruption and demyelination alterations. Furthermore, a study has reported two cases of patients presenting with only DWI and FLAIR high signal in the corpus callosum in the early stages of the disease, which may be an early indicator for the diagnosis of NIID (Wang et al., 2020). We noted significant reductions in MK, RK, AK, KFA, and FA values, along with prominent increases in the MD values within the corpus callosum’s white matter fibers in patients with NIID. These alterations suggest disruptions in myelin structure or integrity, indicating similar susceptibility in callosal commissural fibers and subcortical arcuate fibers. Notably, a study investigating patients with essential tremors observed a positive correlation between microstructural abnormalities in the right superior cerebellar peduncle and tremor severity (Lu et al., 2023). We postulated that the reduced KFA and FA values and elevated MD in the cerebellar peduncle may correlate with clinical manifestations such as tremor and ataxia in patients with NIID, although the exact mechanisms require further exploration. Prior studies have discovered that alterations in WM within the anterior corona radiata are associated with incontinence, its severity, and degree of bother (Zuo et al., 2024; Kuchel et al., 2009), which may also provide a novel viewpoint to explain the prevalence of dysuria symptoms in patients with NIID. These findings emphasize the potential of white matter fibers diffusion properties as a promising biomarker for the diagnosis of NIID. Furthermore, our study demonstrated that DKI parameters exhibited greater sensitivity than DTI parameters in detecting brain tissue integrity damage in NIID patients. This may be attributed to the fact that DKI is more adept at describing the intricate cross-talk of nerve fibers within the brain (Marrale et al., 2016).

With respect to the clinical relevance of WM abnormalities, we found strong correlations between the DKI parameters of RLIC_R, PTR_R, and UNC and neuropsychological assessments. Classified as projection fibers, the internal capsule and posterior thalamic radiation are implicated in attention-related disorders (Schmahmann et al., 2008). Moreover, the uncinate fasciculus represents the most substantial bundle of fronto-temporal fibers. It connects various parts of the prefrontal cortex to the anterior temporal lobe, including critical regions like the hippocampus and amygdala. These correlations suggest that NIID-associated cognitive decline may result from microstructural alterations, such as axonal loss or demyelination within these tracts.

Prior studies on the neurologic functions of NIID patients have yielded inconsistent results. One report documented decreased ASL perfusion during acute stroke-like episodes in a single NIID case, followed by increased perfusion post-episode (Fujita et al., 2017). Conversely, other research found no ASL perfusion abnormalities in NIID patients (Chen et al., 2018). Additionally, analyses using MRS and PET-CT indicated reduced NAA/Cr ratios and glucose metabolism bilaterally, suggesting neuronal dysfunction in NIID, potentially linked to cognitive impairment (Liu et al., 2019). In this study, we employed rs-fMRI to investigate the functional changes in the brains of NIID patients for the first time. Interestingly, we identified a significant reduction in functional connectivity among several critical brain networks in patients with NIID compared to HCs. The disrupted connectivity between the aDMN and SMN might underlie the motor deficits characteristic of NIID, including challenges in fine motor control and coordination. Reduced connectivity between the SMN and SN might suggest difficulties in detecting, integrating, and responding to relevant sensory stimuli, potentially leading to observed deficits in attention and responsiveness in NIID patients (Menon and Uddin, 2010). These alterations in network connectivity highlight the widespread impact of NIID on brain function, which may provide new insights into the neuropathological basis of NIID and may guide the development of targeted therapeutic strategies aimed at restoring network connectivity and ameliorating clinical symptoms.

Despite the promising results, there were some limitations in this study. Firstly, the sample size of this study was small because of the low incidence rate of NIID and it was a cross-sectional study. Further longitudinal studies with larger cohorts are required to substantiate these findings and further explore the relationship between imaging parameters and the copy numbers of *NOTCH2NLC* GGC in NIID. Furthermore, concerning neuropsychological assessment, we exclusively employed the MMSE and MoCA scores to evaluate the subjects’ cognitive abilities. Although these instruments offer significant insights, they might not fully capture every dimension of cognitive deterioration. Lastly, we did not categorize patients with NIID into different subtypes based on clinical presentations due to the small sample size, and we will investigate the imaging characteristics of NIID patients with different clinical subtypes in future studies.

## Conclusion

In summary, significant impairment in microstructural and functional connectivity in patients with NIID were investigated, and these changes are closely associated with neuropsychological deficits. Our results highlight the potential of advanced imaging techniques such as DKI as neuroimaging biomarkers for the detection and monitoring of NIID, contributing to a deeper understanding of the pathological mechanisms of NIID.

## Data Availability

The raw data supporting the conclusions of this article will be made available by the authors, without undue reservation.

## Footnotes

^1^ http://www.fmrib.ox.ac.uk/fsl

^2^DKE, http://www.nitrc.org/projects/dke

^3^ http://www.fil.ion.ucl.ac.uk/spm

^4^ http://www.restfmri.net/forum/index.php

^5^DPARSF, http://rfmri.org/DPARSF

^6^GIFT, http://ictab.sourceforge.net/
