# Supplementary material for: Diffusion and functional MRI reveal microstructural and network connectivity impairment in adult-onset neuronal intranuclear inclusion disease
Source: Front Aging Neurosci. 2024 Oct 11;16:1478065. doi: 10.3389/fnagi.2024.1478065 (PMC11502314; doi:10.3389/fnagi.2024.1478065)
Supplement: Supplementary file 1 [file Table_1.docx]

**Supplementary Table S1.** Tract-based spatial statistics (TBSS) results in white matter regions of DKI and DTI metrics in the NIID patients.

| Indexes | Voxels | *t* value | The percentage of the abnormal voxels |
| --- | --- | --- | --- |
| DKI_MK | 53818 | -3.4156 | 54.3% |
| DKI_RK | 65425 | -3.7296 | 66.0% |
| DKI_AK | 18597 | -3.1422 | 18.8% |
| DKI_KFA | 92511 | -4.3849 | 93.4% |
| DKI_FA | 93194 | -5.4651 | 94.1% |
| DKI_MD | 91603 | 4.6532 | 92.4% |
| DTI_FA | 74199 | -3.7757 | 63.2% |
| DTI_MD | 55246 | 3.4208 | 31.0% |

Corrected by threshold-free cluster enhancement (TFCE) criterion and set at *p* < 0.05.

DKI: Diffusion Kurtosis Imaging; DTI: Diffusion Tensor Imaging; NIID: Neuronal Intranuclear Inclusion Disease; MK: Mean Kurtosis; RK: Radial Kurtosis; AK: Axial Kurtosis; KFA: Kurtosis Fractional Anisotropy; FA: Fractional Anisotropy; MD: Mean Diffusivity.
