# Supplementary material for: Diffusion and functional MRI reveal microstructural and network connectivity impairment in adult-onset neuronal intranuclear inclusion disease
Source: Front Aging Neurosci. 2024 Oct 11;16:1478065. doi: 10.3389/fnagi.2024.1478065 (PMC11502314; doi:10.3389/fnagi.2024.1478065)
Supplement: Supplementary file 2 [file Table_2.docx]

**Supplementary Table S2.** The relationship between the copy numbers of *NOTCH2NLC* GGC and DKI parameters in abnormal brain regions of GM and WM in the NIID patients.

| Indexes | *r* | *p* value |
| --- | --- | --- |
| GM_MK | -0.212 | 0.430^*^ |
| GM_RK | -0.225 | 0.402^#^ |
| GM_AK | -0.079 | 0.772^#^ |
| GM_KFA | 0.206 | 0.444^*^ |
| WM_MK | -0.280 | 0.293^#^ |
| WM_RK | -0.271 | 0.311^#^ |
| WM_AK | -0.257 | 0.337^#^ |
| WM_KFA | 0.292 | 0.273^*^ |
| WM_FA | -0.184 | 0.496^#^ |
| WM_MD | 0.029 | 0.914^*^ |

Pearson's correlation analysis (^#^) was employed for data that conformed to a normal distribution, while Spearman's correlation analysis (^*^) was used for non-normally distributed data.

DKI: Diffusion Kurtosis Imaging; GM: Gray Matter; WM: White Matter; NIID: Neuronal Intranuclear Inclusion Disease; MK: Mean Kurtosis; RK: Radial Kurtosis; AK: Axial Kurtosis; KFA: Kurtosis Fractional Anisotropy; FA: Fractional Anisotropy; MD: Mean Diffusivity.
