# Supplementary material for: Diffusion and functional MRI reveal microstructural and network connectivity impairment in adult-onset neuronal intranuclear inclusion disease
Source: Front Aging Neurosci. 2024 Oct 11;16:1478065. doi: 10.3389/fnagi.2024.1478065 (PMC11502314; doi:10.3389/fnagi.2024.1478065)
Supplement: Supplementary file 3 [file Data_Sheet_1.docx]

**
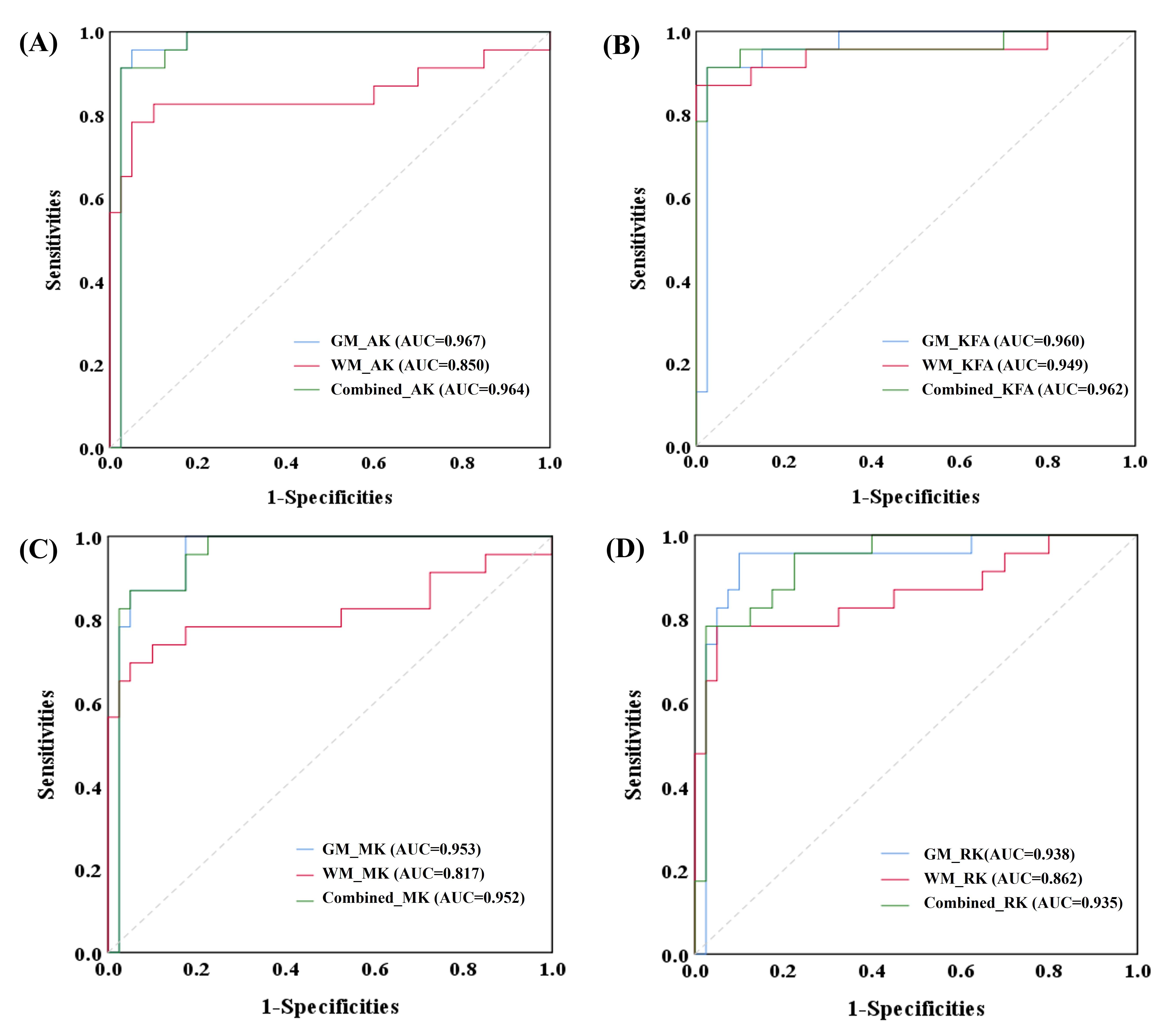
**

**Supplementary Figure 1** Diagnostic efficacy of MK, RK, AK, and KFA metrics in GM, WM and combined regions in identifying NIID and healthy controls.

MK: Mean Kurtosis; RK: Radial Kurtosis; AK: Axial Kurtosis; KFA: Kurtosis Fractional Anisotropy; GM: Gray Matter; WM: White Matter; NIID: Neuronal Intranuclear Inclusion Disease; AUC: Areas under the ROC curve.
